# Out-of-pocket expenditure and catastrophic costs due to COVID-19 in Indonesia: A rapid online survey

**DOI:** 10.3389/fpubh.2023.1072250

**Published:** 2023-03-24

**Authors:** Firdaus Hafidz, Insan Rekso Adiwibowo, Gilbert Renardi Kusila, Mahlil Ruby, Benyamin Saut, Citra Jaya, Wan Aisyiah Baros, Dedy Revelino, Erzan Dhanalvin, Ayunda Oktavia

**Affiliations:** ^1^Department of Health Policy and Management, Faculty of Medicine, Public Health, and Nursing, Universitas Gadjah Mada, Yogyakarta, Indonesia; ^2^Badan Penyelenggara Jaminan Sosial Kesehatan, Jakarta, Indonesia

**Keywords:** COVID-19, out-of-pocket expenditure, catastrophic costs, Indonesia, online survey, socioeconomic impact

## Abstract

**Background:**

The Corona Virus Disease 2019 (COVID-19) pandemic has created a substantial socioeconomic impact, particularly in developing countries such as Indonesia.

**Purpose(s)/objective(s):**

This study aimed to describe the COVID-19-related out-of-pocket spending of Indonesian citizens and the proportion of whom experienced catastrophic health spending during the COVID-19 pandemic using the patient's perspective.

**Methodology:**

We conducted a rapid cross-sectional online survey across provinces in Indonesia to capture participants' experiences due to COVID-19. Data were collected between September 23rd to October 7th of 2021 including demographics, income, and expenditures. Descriptive statistics were used to analyze the respondents' characteristics. Patients's perspective of total cost was estimated from out-of-pocket of COVID-19 direct costs and compared them to total expenditure. If the proportion of COVID-19 total costs exceeded 40% of the total expenditure, the respondents were deemed to have faced catastrophic costs.

**Results:**

A total of 1,859 respondents answered the questionnaire. The average monthly income and expenditure of respondents were 800 USD, and 667 USD respectively. The monthly expenditure was categorized into food expenditure (367 USD) and non-food expenditure (320 USD). The average of COVID-19-related monthly expenditure was 226 USD, including diagnostic expenditure (36 USD), preventive expenditure (58 USD), medical expenditure (37 USD for COVID-19 treatment; and 57 USD for post-COVID-19 medical expenses), and non-medical expenditure (30 USD). Analysis showed that 18.6% of all respondents experienced catastrophic costs while 38.6% of the respondents who had COVID-19 treatment experienced catastrophic costs.

**Conclusion:**

The high proportion of catastrophic costs among respondents suggests the need for COVID-19 social protection, especially for COVID-19 diagnostic and prevention costs. The survey findings have led the government to increase the benefit coverage other than medical costs at the hospitals.

## 1. Introduction

Nobody ever doubts that health is an indispensable human right. For that, every human being should have an equitable opportunity to reach their optimal health capacity despite their age, gender, geographical location, sociocultural background, and financial state. The importance of health then held a pivotal role for the United Nations to declare the Sustainable Development Goals (SDGs) in the year 2015 (1). Since then, they have performed as a catalyst in global development efforts.

However, achieving the ambitious SDGs does not come without any obstacles. Only 5 years after the SDGs have been widely implemented, the COVID-19 pandemic destabilized the lives of billions of people. Economic turbulence, mobilization restriction, a global shortage of medical resources, and political tensions are inevitable following the years of the pandemic (2, 3).

The Indonesia Statistic Center Agency showed that the number of people living in poverty in Indonesia has increased to 10.19% of the total population in 2020, with higher poverty found in the non-urban areas (4). Besides, the World Bank reported that around 97 million additional people have fallen into poverty due to the pandemic (5). However, the number could be far underestimated because the expenditure baseline was not regularly updated with the country's inflation rate. Moreover, many households overcame the financial shock by altering their daily food consumption and re-engaging with agricultural production, resulting in their actual financial condition during the surveys being masked (5, 6).

This financial impact can threaten health-seeking behavior, including those who have been covered with social health insurance. Lower-income insurance holders still experienced less time with the health workers, longer waiting times for medical procedures, and more non-health related burdens (transportation, accommodation, working permission, etc.) for accessing healthcare (7). Additionally, families and individuals need to allocate more money as a consequence of the COVID-19 preventive measures enforcement. As an illustration, a nuclear family in Vietnam spent 20% of their monthly income on disposable facemasks alone (8).

The pandemic also restricts patient-health workers' face-to-face contact. This does not only prevent them from receiving complete examinations and treatments. This limitation is also deteriorating medication adherence among patients with chronic illness, particularly those with higher psychological stress resulting from the pandemic (9, 10). Furthermore, many underinsured patients face a routine drug shortage. A study among people with diabetes in Indonesia showed that 56% of them obtain their drugs from the out-of-pocket (OOP) scheme during the pandemic (11). Nevertheless, Komazawa et al. found that 12% of the Indonesian elderly had no purchasing power to do so (12). This condition was worse among the patients who required companionship to access healthcare because an additional single companion meant more spending to reach the health services needed (12).

The OOP health spending during the pandemic should also be a reason for concern. Many remain unrecorded since this spending could be done outside the appointed healthcare providers due to the increasing self-medication (13). The patients with COVID-19 and those suffering from long-COVID symptoms were not excluded from this practice (14). The unprescribed antibiotic, corticosteroid, and antiviral use were widely found among them (15, 16).

From Indonesia's perspective, the government has classified the COVID-19 pandemic as a non-natural disaster, hence all mitigation endeavors and treatment costs are imposed on the state budget regardless of the insurance ownership status (17, 18). However, the collapse of the healthcare system, creating anxiety about contracting COVID-19, the inconclusive clinical trial that spread throughout social media, and dissatisfaction with the previous treatments have led more people to arrange their own treatment plans (19, 20). Besides, the extra spending also occurs from the COVID-19 diagnostic testing and the preventive measures that become a compulsory item for people's mobilization and public gatherings (21).

The OOP pattern especially among COVID-19 patients therefore revealed the paradox in our health system. When the financial support does not meet the infrastructure's preparedness, even people under various universal health coverage schemes will flee to the available OOP services. This OOP option hence should raise the concerns for financial protection, especially since the pandemic has forced the prices to escalate which may lead to further financial catastrophes (22). To our knowledge, no study has explored Indonesia's individual expenditures related to the COVID-19 pandemic. Its impacts on the household and personal economic condition are also unknown. On that account, this study then aimed to investigate how those spendings may be inescapable to be major challenges for the SDGs indicator number 3.8, which mentioned: “*Achieve universal health coverage, including financial risk protection, access to quality essential health-care services and access to safe, effective, quality and affordable essential medicines and vaccines for all*.” (1).

## 2. Methods

### Research design

A rapid cross-sectional survey was conducted for a two-week period from 23rd September 2021 to 7th October 2021. Due to the COVID-19 pandemic restrictions, the survey was done through an online platform called SurveyMonkey (http://www.surveymonkey.com). The data were collected using a structured questionnaire aimed to capture the experience of the Indonesian population regarding the COVID-19 pandemic. Only participants with Indonesia's citizenship, as young as 18 years old or older, and having no health-related backgrounds (doctor, nurse, midwife, etc.) could be included. This study is approved by the Medical and Health Research Ethics Committee of the Faculty of Medicine, Public Health, and Nursing, Universitas Gadjah Mada, Yogyakarta, Indonesia with the approval number: KE/FK/0945/EC/2021.

The survey covered a target sample size of 1,068 respondents, which was calculated by a software called Raosoft. This number was obtained after deciding the desired 95% confidence level with a margin of error as low as 3%. Furthermore, the number of populations to be represented was determined from the Indonesian population of 272,229,372 people with 50% response distribution. To reach the number of minimal respondent's, the convenience sampling method was used. Every regional office of BPJS Kesehatan in 33 Indonesia's provinces was involved to distribute the survey link to the general public. The broadcast mainly utilized the chat groups in various social media platforms such as Whatsapp, Facebook, Telegram, and Instagram.

### Instrument development

The statements and questions of the survey were written in standardized Indonesian language. Before the respondents were entered to the survey's questions sections, they were firstly informed about the aim of the survey, the researchers, the methodology, the confidentiality of the data, and how the result would be published. Only those who fully consent to agree to be involved would be directed to questions sections.

The first part of the questionnaire was the demographic information. The gender, age, marital status, last formal education, and their occupation were asked in this section.

The second part of the questionnaire aimed to gather health insurance data. This part consisted of the national health insurance (NHI) participation under the BPJS Kesehatan, type of NHI membership, and class of NHI membership.The participation was categorized into active, inactive, and not participated. We included four categories of insurance membership type that were acknowledged by the BPJS Kesehatan: non-worker (Non Penerima Bantuan Iuran- Bukan Pekerja), worker-without salary scheme (Pekerja Bukan Penerima Upah), worker-with salary scheme (Pekerja Penerima Upah), and subsidized scheme (Penerima Bantuan Iuran). Worker-without salary participants were the insurance members, whose work did not require an employment bond. The contrary was applied to the worker-with salary scheme. Furthermore, the non-workers group is specified insurance for the financial investors, employers, retirees, and military veterans. These three categories have the obligation to pay a monthly fee. On the other hand, the insurance for the subsidized scheme group is fully covered by state funds and they had no responsibility to pay the monthly insurance fee.

The insurance holders were also classified to different classes of service (1st, 2nd, 3rd class) which depended on the monthly insurance fee. This class will determine the inpatient service at the secondary care. However, those coming from the subsidized category will automatically receive the 3rd class.

The third part of the questionnaire was the health status which covered their COVID-19 history and vaccination status. This history was classified to: has ever confirmed, suspected to contract COVID-19 but was not tested, suspected to contract COVID-19 and the test resulted negative, has never been confirmed or suspected, and did not know the history. The vaccination status was also gathered, whether the public has already received the first, second, or has not been vaccinated.

Economic status was also asked in this study, which was included in the fourth section of the questionnaire. The household's income was explored among the respondents. The expenditure was grouped into two different themes: the monthly household expenditure and the individual's COVID-19 related expenditure. The monthly household expenditure consisted of the money spent on food, non-food (transportation, electrical bill, leisure, etc), and their overall monthly expenditure (including tax, insurance payment, education tuition, etc). Moreover, the respondents were specifically asked about their direct medical cost, which expenditures related to the COVID-19 pandemic including diagnostic test, prevention, acute treatments, and post-COVID-19 rehabilitation in the last 1 month. The period of 1 month was chosen to minimize the recall bias among the respondents. All the numbers were answered by the respondents in Indonesia's currency (IDR-rupiah). For this paper, we put the amount as in USD (USD1 = IDR15,000).

### Analysis

Responses from the online survey were stored in a cloud system managed by the BPJS Kesehatan and the researchers of this study. To open the data, a specific institutional username was used to limit the access. The analysis was performed using R version 4.0.4 (R Foundation for Statistical Computing, Vienna, Austria). This study conducted a descriptive statistics analysis. The demographics, NHI, health status, and economic information of the respondents were presented in frequencies and proportions. The patients' perspective of total COVID-19 OOP proportion expenses was estimated from each individual COVID-19 related spending and comparing them to their average monthly expenses (Equation 1). When this proportion exceeded >40%, they were categorized to have catastrophic health spending. The focus on the 40% threshold was obtained from the catastrophic OOP study conducted by World Health Organization (23). The number of respondents experiencing catastrophic was compared to the total respondents to obtain the proportion of catastrophic (Equation 1).

Equation 1. The proportion of catastrophic and COVID-19 proportion expenses.


$$\begin{array}{l} The\;proportion\;of\;catastrophic\;=\\ \frac{Number\;of\;respondents\;with\;COVID19\;proportion\;expenses\;>\;40\%}{Total\;respondents}\\ Individual\;COVID19\;proportion\;expenses\;=\\ \;\frac{COVID\;19\;related\;expenses}{Average\;monthly\;expenses}\;\times 100 \end{array}$$

## 3. Results

A total of 1.859 respondents were included in this study. The general characteristics of the study participants are shown in Table 1. The gender distribution of participants was roughly equal (45% for male vs. 55% for female), with most of the participants (85%) were between 18 and 39 years old. The participants were mostly well educated, with 1,577 (84.7%) receiving some form of higher education. A total of 1,048 (56.5%) participants claimed to never have been infected or did not know whether they were ever infected with COVID-19. Remarkably, it was found that as high as 32.6% of the participants had ever tested positive for COVID-19. Furthermore, the majority of our study participants (93.3%) had received the 2nd vaccination dose.

**Table 1:** General characteristic of study participants.

**Characteristic**	***N* = 1,859^a^**
**Gender**	
Male	832 (45%)
Female	1,027 (55%)
**Age group**	
18–29	766 (41%)
30–39	822 (44%)
40–49	229 (12%)
50–59	42 (2.3%)
≥60	0 (0%)
**Marital status**	
Unmarried	528 (28%)
Divorced and not remarried	28 (1.5%)
Widowed and not remarried	10 (0.5%)
Married	1,293 (70%)
**Education level**	
Junior high school or equal	6 (0.3%)
Senior high school or equal	275 (15%)
Diploma and undergraduate	1,433 (77%)
Postgraduate	144 (7.7%)
**Occupation**	
Civic worker	74 (4.0%)
Employees	1,446 (77%)
Laborer (including farmer)	19 (1.0%)
Academic work (researcher, lecturer, teacher, student)	28 (1.5%)
Entrepreneur	38 (2.0%)
Freelancer	12 (0.6%)
Pension/no work	20 (1.1%)
Others	222 (11.9%)
**National insurance participation**	
Active member	1,828 (98%)
Inactive member	27 (1.4%)
Not participated	4 (0.2%)
**Type of insurance membership**	
Contributory-non worker	26 (1.4%)
Worker-non salary system	48 (2.6%)
Worker- with salary system	1,735 (94%)
Non-contributory	31 (1.7%)
**Class of insurance membership**	
1st	1,523 (83.0%)
2nd	270 (15.0%)
3rd	46 (2.5%)
**COVID-19 status**	
Ever confirmed	606 (32.6%)
Suspected, not tested	57 (3.1%)
Suspected, tested negative	148 (8.0%)
Never been confirmed or suspected	904 (48.7%)
Not know	144 (7.8%)
**Vaccination status**	
Not yet	58 (3.2%)
1st dose	67 (3.6%)
2nd dose	1,732 (93.3%)

Here, almost all (98%) participants were members of the National Health Insurance, with the largest proportion coming from the worker-with salary group (94%) and holding the 1st class membership (83%).

The average monthly income of the respondent's household was 800 USD, with a median of 690 USD, while the overall monthly household expenses (both food and non-food) was 667 USD (367 for food; 320 for non-food). The average monthly individual expenses of COVID-19 related expenditure averaged 226 USD, further classified into expenses for diagnostic tests (36 USD), preventive measures such as masks, hand sanitizers (58 USD), acute treatment of those infected with COVID-19 (37 USD) and treatment of post-COVID-19 health complaints (57 USD). Those expenses are listed in Table 2.

**Table 2:** Monthly income and expenses of the study participants.

**Type**	**Average (median) USD**
Monthly household income	800 (690)
**Monthly household expenditure**	
Food	367 (206)
Non-food	320 (276)
Overall monthly expenses	667 (551)
**Past month individual COVID-19 related expenditure**	
Diagnostic test (PCR, antigen, etc)	36 (11.0)
Prevention (mask, hand sanitizer, etc)	58 (20.7)
Acute treatment	37 (17.2)
Post-COVID rehabilitation	57 (37.7)
Overall COVID-19 expenses	226 (137.9)

**Figure 1:**
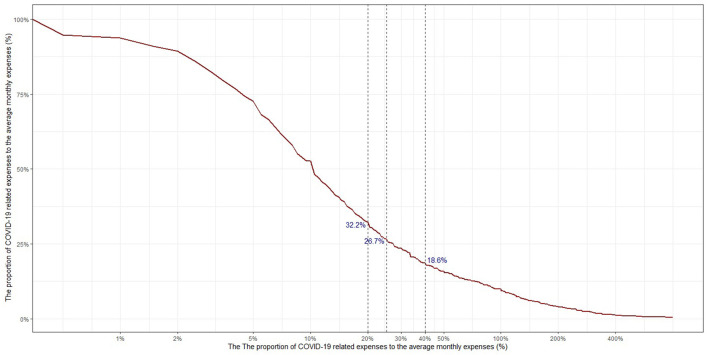
The proportion of individuals who experienced catastrophic health spending related to COVID-19 expenses.

Figure 1 shows the proportion of COVID-19 related expenses to the average monthly expenditure of all respondents. Based on the 40% threshold used in this study to define catastrophic health expenditure, 18.6% of the participants experienced the catastrophic costs. To explore the impact, two additional thresholds were applied. The 20 and 25% thresholds resulted in 32.2 and 26.7% of the respondents to be affected by the COVID-19 catastrophic cost respectively.

**Figure 2:**
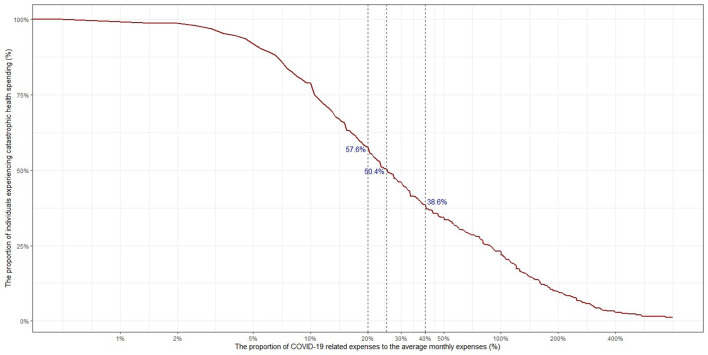
The proportion of individuals who had out-of-pocket COVID-19 treatments in the past month that experienced catastrophic health spending.

We then adjusted the calculation only to the respondents receiving OOP COVID-19 treatments in the past month (as seen in Figure 2). These treatments could be either acute or the rehabilitation procedures. Among these patients, 38.6% of the participants were hit by catastrophic health expenditure. If 20% threshold were applied, there were 57.6% participants experiencing the catastrophic event. When 25% of the monthly expenses went to COVID-19 related expenses, 50.4% respondents were financially affected.

## 4. Discussion

The average monthly household income among the respondents in this study was USD800. If their household is composed of two working adults, the estimation of their average individual monthly income was USD400. This was slightly higher compared to Indonesia's per capita income extracted from the year 2021 gross domestic product (GDP) resulting in USD 362/month (24). However, there were respondents whose income was below that amount. Moreover, a consumer survey from PricewaterhouseCoopers (PwC) Indonesia found a 65% decline since COVID-19 was first detected (25).

Our study found that the average COVID-19 preventive measure contributed the highest in COVID-19-related purchases (USD 58). It was then calculated that an average of 7.25% of household income was lost for the preventive measures alone. This number was higher compared to the same expenditure in Malaysia with USD 46 or 4.3% of household income (26). This Malaysian survey even included supplements and complementary medicine as part of the preventive measure, while ours separated the use of any taken medication in OOP treatments.

Furthermore, the COVID-19 cost per capita across 22 low-middle-income countries ranges from USD43.39 to USD75.57 without including the possible OOP expenditure. The cost absorbed was up to 12% of countries' GDP (27). A study in India showed the difference in OOP expenditure between public and private hospitals for COVID-19 hospitalization, which then translated to around 3% of hospitalizations in public hospitals and 59% in private hospitals resulting in catastrophic expenditure (28). Our study may have a higher catastrophic estimation with 79% who may have experienced catastrophic health expenditure because we did not only limit the OOP from hospitalization alone.

Although this study did not explore whether the respondents practiced self-medication, it is still believed that the amount of expenditure mentioned by them also contained the over-the-counter products. Besides, the advantageous claims of various medications have been widely accepted by Indonesian society, although the information often came from relatives, social media, or even product brochures (29). The Indonesian behavior to purchase and store traditional medicine may also lead to even greater OOP (30). On top of that, drug hoarding behavior was found among households during the pandemic (31). Since the data in this study were collected immediately after Indonesia was terribly hit by the Delta variant wave, there was a probability that the definition of COVID-19 expenses “within the past month” may include the excessive drug stockpiling behavior, marking the high OOP expenditure in this study. This aspect then should be further investigated whether Indonesia's citizens are regularly purchasing unintended medications or only limited to a one-time shock panic buying. Hence, the period, duration, and fluctuation of the financial impact of the practice may be better understood.

Moreover, the second largest component of COVID-19-related expenditure was rehabilitation treatment (USD 57). A study conducted by Iannaccone et al. in Italy found that COVID-19 rehabilitation was twice higher compared to similar rehabilitation procedures during the pre-pandemic period. However, the study also mentioned that the expenses increased due to the additional health professionals needed although the number of in-site beds decreased (32). Nonetheless, a study in Switzerland found that a third of post-COVID-syndrome patients who did not fully return to their prior normal health did not seek any medical support, indicating that the relevance of post COVID-19 care is widely scattered and really depends on individuals' health perception (33). Therefore, it should be further explored whether the post-pandemic rehabilitation treatment cost will be as high as the cost during the pandemic era for those who have or have not contracted COVID-19. How the rehabilitation resources should be distributed or invested to a greater extent and the insurance scheme for rehabilitation purposes should also be provided.

The importance of stakeholders' involvement and healthcare benefits understanding is largely needed to mobilize adequate financing resources to the post-pandemic health coverage systems. However, to meet these conditions, the health insurance scheme has to continue to evolve by adapting to the environment, demographic, epidemiological, cultural, and technological changes (34).

In these circumstances, endemic diseases that have not been successfully eradicated lead to financial catastrophe. One of the most prominent is tuberculosis which creates a 36% household catastrophe. The number is even higher among poor households (43%) and surged with multi-drug-resistance cases (83%) (35). A systematic review from Ghazy et al. also found drug sensitivity and co-infection to be the major predictor for tuberculosis-related financial catastrophe (36). Here, a line between the OOP antibiotic self-medication and antibiotic resistance can be interpreted to jeopardize the community's health and financial status at a more tremendous level. That study also added that active findings of tuberculosis cases formed lower financial catastrophes to the patients (12%), in comparison with the passive ones (30%) (36). Hence, the health system and its financial scheme need to incorporate the role of disease tracing, which fortunately has been applied for COVID-19 pandemic.

Furthermore, Indonesia is facing an undeniable rapid aging population, since it is projected that the elderly will account for 19.7% of the total population by 2045. With 300 thousand decrements in 2045's number of births compared to the year 2020, the dependency ratio of the non-working age population will escalate to 53.4 in 2045 (37). Regarding this aging population, the disability resulting from multiple diseases may burden Indonesia's economic and social landscape. In our study, the high rehabilitation expenses can portray the health-coverage challenges to overcome the disease disability, which is supported by a study in Uganda that discovered a significant association between disability with the household financial risk (38).

Indonesia should understand this growing issue. The health budget should not be static. Indonesia allocated 5% of its state budget in 2019 to cover the national health expenditure (39). Although this percentage has been consistent with the state constitution, this health budget was only 2.9% if it is compared with Indonesia's GDP. This percentage is lower than Thailand (3.79%), Vietnam (5.25%), and the Philippines (4.08%) (40). The macro-fiscal policies and tax revenue mobilization should be altered with the health service demands (41). Generating higher domestic revenue and better healthcare-stakeholders governance are suggested to improve the public expenditure on health (42).

Lastly, the OOP expenses can be prevented by strengthening disease surveillance. During the crisis period, this effort must be incorporated into the national and regional disaster risk management system. This should include the fast response in an epidemiological study, financing, infrastructure, health workers' resources, and health supplies (43).

### Limitation of the study

This study was performed without no limitations. Firstly, We acknowledge that the respondents of this study were very likely to live in an area with a well-developed internet connection, have better internet or online platforms literacy, and have more concern or interest in COVID-19 issues. These factors can explain why the respondents of this study came from better education backgrounds, relatively higher incomes, and worked as an employee or white-collar worker. Since the survey itself was distributed using various online platforms, the willingness to answer the survey was then influenced by the time the survey's link broadcast has been received, reminders from the survey's broadcasters or distributors, social networks, and many other factors. Even though they could not fully represent the larger Indonesian population, the economic catastrophe proven by this study could figure out how the COVID-19 pandemic could have a large impact even on the relatively well-established society. This could then possibly be translated to more serious economic impacts that have been experienced by the underprivileged groups which have not been included. As the social restrictions are slowly loosened, more studies with better approaches should be conducted to investigate how COVID-19 and its health-related expenditure had affected different communities. This will be important to generate extensive understanding to act as evidence for the anticipated health and economic related policies. This study could serve as insight and a catalyst for more similar studies to be generated in the near future.

## 5. Conclusion

To determine the preparedness of the UHC to cover the COVID-19 pandemic, three basic factors need to be evaluated; the OOP spendings to overcome the disease, the benefit packages, and the population coverage. The high proportion of catastrophic costs among respondents due to various OOP expenditures suggests the need for further COVID-19 social and health protection beyond the disease treatments. The stakeholders need to consider the benefit package for an extensive public health issue such as the COVID-19 pandemic, whose prevention and post-acute services lead to mandatory individual purchase. NHI must be adaptive in generating and distributing the health funding based on the scientific-based healthcare demands, especially during the crisis period. Strengthening the disaster management responses with adequate scientific-based information is vital to minimize the financial catastrophic cost.

## Data availability statement

The raw data supporting the conclusions of this article will be made available by the authors, without undue reservation.

## Ethics statement

The studies involving human participants were reviewed and approved by Medical and Health Research Ethics Committee of the Faculty of Medicine, Public Health, and Nursing, Universitas Gadjah Mada, Yogyakarta, Indonesia with the approval number: KE/FK/0945/EC/2021. The patients/participants provided their written informed consent to participate in this study.

## Author contributions

FH, IA, and GK: conceptualization, methodology, survey development, data management, data analysis, writing—original draft preparation, writing—review, and editing. AO and ED: data collection, data management, writing—review, and editing. MR, BS, CJ, DR, and WB: conceptualization, methodology, survey development, data collection, writing—review, and editing. All authors contributed to the article and approved the submitted version.
